# Process-Based Modeling of Phenology and Radial Growth in *Pinus tabuliformis* in Response to Climate Factors over a Cold and Semi-Arid Region

**DOI:** 10.3390/plants13070980

**Published:** 2024-03-29

**Authors:** Zihong Man, Junzhou Zhang, Junjun Liu, Li Liu, Jiqin Yang, Zongying Cao

**Affiliations:** 1 Gansu Liancheng Forest Ecosystem Field Observation and Research Station, Lanzhou University, Lanzhou 730333, China; 2 Liancheng National Nature Reserve in Gansu, Lanzhou 730300, China; 3 Key Laboratory of Western China’s Environmental Systems (Ministry of Education), College of Earth and Environmental Sciences, Lanzhou University, Lanzhou 730000, China

**Keywords:** tree-ring, climate change, Vaganov–Shashkin (VS) model, phenology, radial growth, Chinese pine

## Abstract

(1) Background: Climate change significantly impacts the phenology and dynamics of radial tree growth in alpine dryland forests. However, there remains a scarcity of reliable information on the physiological processes of tree growth and cambial phenology in response to long-term climate change in cold and semi-arid regions. (2) Methods: We employed the process-based Vaganov–Shashkin (VS) model to simulate the phenology and growth patterns of Chinese pine (*Pinus tabuliformis*) in the eastern Qilian Mountains, northeastern Tibetan Plateau. The model was informed by observed temperature and precipitation data to elucidate the relationships between climate factors and tree growth. (3) Results: The simulated tree-ring index closely aligned with the observed tree-ring chronology, validating the VS model’s effectiveness in capturing the climatic influences on radial growth and cambial phenology of *P. tabuliformis*. The model outputs revealed that the average growing season spanned from mid-April to mid-October and experienced an extension post-1978 due to ongoing warming trends. However, it is important to note that an increase in the duration of the growing season did not necessarily result in a higher level of radial growth. (4) Conclusions: While the duration of the growing season was primarily determined by temperature, the growth rate was predominantly influenced by water conditions during the growing season, making it the most significant factor contributing to ring formation. Our study provides valuable insights into the potential mechanisms underlying tree growth responses to climate change in cold and semi-arid regions.

## 1. Introduction

Radial tree growth (wood formation), which is the product of cambial cell division and differentiation [1], represents the most important way for long-term carbon sequestration in forests [2,3]. However, the increasing air temperatures and shifting precipitation patterns present a challenge to this carbon sequestration capacity by affecting various physiological processes [4], including decreasing growth rates [5,6] and shifts in the growth phenology of both leaves and cambia [1,7]. Given the projected climate changes expected in the coming decades, it is essential to assess long-term growth phenology and dynamics in response to climate to predict and quantify the future carbon sequestration potential.

To assess growth phenology and seasonal radial growth of trees, researchers employ methods such as monitoring of microcoring and simulation of physiological tree growth models. Microcoring provided a continuous and direct record of phenology and growth dynamics, facilitating the elucidation of uncertainties in radial tree growth at the cellular level, which is crucial for understanding the response mechanism between climate and tree-ring formation [8,9]. However, the labor-intensive nature of direct monitoring constrains its utility for long-term phenological and growth trend analysis [9]. Therefore, there has been a shift towards the development and application of process-based physiological tree growth models to address these challenges [10,11].

The Vaganov–Shashkin (VS) model is one of the most efficient and widely used physiological models for verifying the relationships between tree growth and climate factors [12]. It outputs growth parameters such as cambial phenology and growth rates, which are instrumental in assessing long-term changes over the long-term study period, typically defined by the span of available climate data or tree-ring indices [11,12]. The VS model has proven its precision in simulating tree growth processes across various regions and has provided reliable growth parameters, forming an important foundation for explicating the physiological mechanisms underlying regional tree growth in response to climate changes [13,14,15,16,17,18,19,20]. Its reliability in simulating and evaluating climate–growth relationships across diverse regions and environmental conditions is well documented [17,21,22].

Semi-arid alpine forests, as indicators of global change, are pivotal for soil and water conservation and carbon sequestration in semi-arid regions [23]. Their sensitivity and vulnerability to climate change necessitate a focus on the growth phenology and radial growth of trees in these forests under the background of climate change, as well as an evaluation of their growth responses and adaptations [24]. The Qilian Mountains are a critical area for examining the effects of climate change on semi-arid alpine forests. This region has experienced continuous warming and a notable increase in the frequency of extreme climatic events [25]. Chinese pine (*Pinus tabuliformis*), the dominant coniferous species of northern China, reaches its westernmost distribution in the eastern Qilian Mountains. Previous studies have shown that the radial growth of *P. tabuliformis* is highly sensitive to climate change and has been widely used to reconstruct precipitation, temperature, and drought indices over the past several hundred years [26,27]. However, although several studies have focused on the phenology and radial growth dynamics of this species using microcoring monitoring [15,28,29], there remains a lack of reliable information on the long-term phenology and growth dynamics of this species in response to climate change.

In this study, we aim to explore the efficacy of the VS model in replicating physiological parameters such as growth phenology and variability patterns of *P. tabuliformis* in the eastern Qilian Mountains over recent decades. Based on previous monitoring studies in this region [29,30], we hypothesize that (i) the onset of radial tree growth is primarily determined by the temperature, while water availability is crucial for the end of tree growth, and (ii) water availability has a more significant impact on growth rates rather than temperature in this semi-arid study region.

## 2. Materials and Methods

### 2.1. Study Region

**Figure 1:**
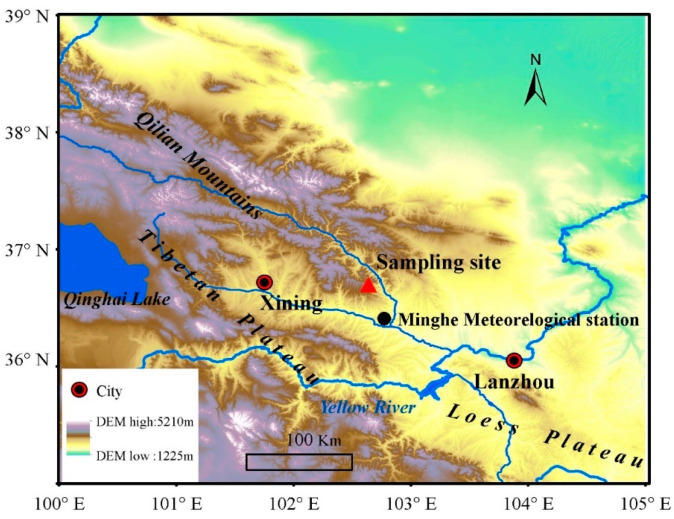
Location of sampling site (red triangle) and nearest national meteorological station (black circle).

Our sampling site was located in the eastern Qilian Mountains, northeastern Tibetan Plateau (36°43′21″ N, 102°37′59″ E, 2070 m asl), marking the transitional zone between the Tibetan Plateau and the Loess Plateau (Figure 1). This area is characterized by pristine mountainous forests with abundant vegetation, displaying a distinct vertical distribution pattern. Within this region, *P. tabulaeformis* is primarily distributed on dry and infertile south-facing slopes below 2600 m above sea level, representing the westernmost boundary of its distribution in China. The sampling site, centrally located along the forest’s edge on a south-facing slope, lies in a remote area with minimal human impact. The vegetation comprises open stands of mono-specific *P. tabulaeformis*, with sparse interspecific competition. All sampled trees are healthy and free from pests and diseases. Given the open canopy of the forest (less than 20% coverage), we expect limited tree-to-tree interactions, with abiotic factors predominantly influencing tree growth.

### 2.2. Meteorological Data

**Figure 2:**
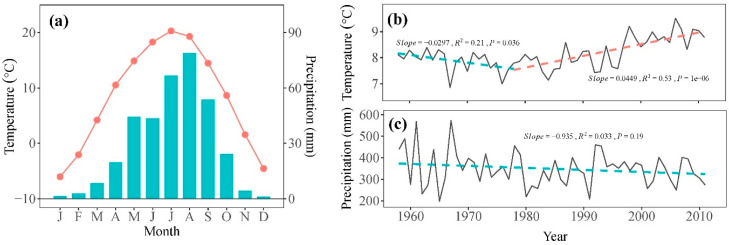
(**a**) The monthly mean temperature (symbol line) and precipitation (bars) during the period 1958–2011 recorded by the Minhe meteorological station. Annual mean temperature (**b**) and total precipitation (**c**) (grey solid line) and their trends (dash line) during the period 1958–2011.

Daily temperature and precipitation data from 1958 to 2011 were sourced from Minhe meteorological station (36°19′48″ N, 102°51′06″ E, 1814 m asl), located approximately 40 km from our sampling site (Figure 1). These data were obtained from the China Meteorological Data Sharing Service System (https://data.cma.cn/, accessed on 18 February 2024) with no missing values. The records indicate a typical semi-arid climate for the study area, with an average annual precipitation of 350.0 mm and an average annual temperature of 8.2 °C for the period of 1958–2011 (Figure 2a). The mean annual temperature showed a significant decreasing trend of 0.30 °C per decade from 1958 to 1978, followed by a significant increasing trend of 0.45 °C per decade from 1978 to 2011 (Figure 2b). Over the study period of 1958–2011, total precipitation exhibited a decrease of 9.35 mm per decade (Figure 2c).

### 2.3. Tree-Ring Data

**Figure 3:**
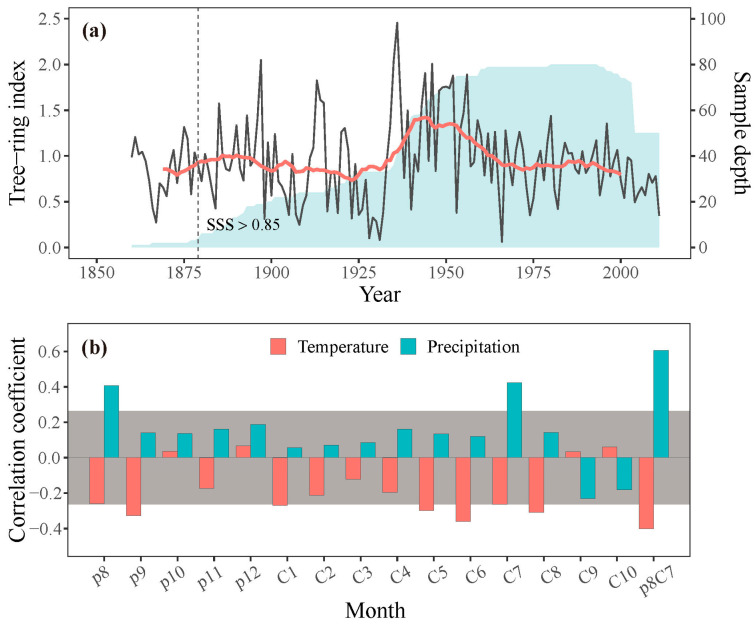
(**a**) Tree-ring width chronology (grey line) with 21-year running averages (red line) and sample depth (shaded area) of the chronology. The dashed line shows the year when SSS exceeds 0.85. (**b**) Correlations between the observed STD chronology and monthly temperature and precipitation; p denotes the previous year (e.g., p8 is the previous August), and C denotes the current year (e.g., C1 is the current January), p8C7 represents the mean value from previous August to current July. Bars surpassing the grey area signify correlations above the 95 % confidence level.

Increment core samples were obtained at breast height (1.3 m) from each tree using increment borers, with two or three samples per tree. These samples were processed in the laboratory, mounted, air-dried, and sanded following standard procedures [31]. Tree-rings were cross-dated and measured to a precision of 0.001 mm using a Velmex measuring system and checked by the COFECHA program for quality control of cross-dating [32]. To remove age-related trends and preserve low-frequency signals, conservative detrending curves such as linear or negative exponential curves were applied to the cross-dated raw indices. The indices were standardized by dividing the raw width of the fitted curve value, then averaged to create standard (STD) chronologies using the ARSTAN program [33]. The representativeness of the common signal in the chronology was assessed by calculating the running mean inter-series correlation coefficient (Rbar) and expressed population signal (EPS) over a 50-year window with 25-year overlaps. The subsample signal strength (SSS), with a threshold value of 0.85, was used to determine the reliable period of the chronology (Figure 3a; Table 1). To examine the characteristics of the STD chronology, parameters such as mean sensitivity (MS), standard deviation (SD), mean inter-series correlation (MR), EPS, and signal-to-noise ratio (SNR) were also calculated (Table 1). The STD chronology was used to analyze the climate–growth relationships and validate the simulation results.

### 2.4. VS Model

The Vaganov–Shashkin (VS) model is a process-based model that operates on several assumptions. Firstly, it posits that cambial activity, and thus the anatomical features of newly formed tracheids, such as radial diameter and cell wall thickness, are primarily influenced by external factors of temperature, photoperiod, and soil moisture. Secondly, the model applies the principle of limiting factors to growth rate calculations, meaning that the growth rate at any given time is constrained by the most limiting factor. Lastly, the VS model simulates variations in growth rate and tree-ring structure in response to current season climatic changes [12].

The model calculates intra-annual radial tree growth as a function of three main factors [12]:

*Gr*(t) = *GrE* (t) × min [*GrT* (t)*, GrW* (t)] (1)

where *GrE* (t), *GrT* (t), and *GrW* (t) represent the partial growth rates on day t of the year, determined independently by solar irradiation (photoperiod), temperature, and soil water content, respectively.

Solar Irradiation: *GrE* is modeled as a harmonic function that considers latitude, declination angle, and hour angles.

Temperature: Growth is influenced by temperature through a piecewise linear function. Growth initiates when accumulated temperature surpasses the threshold value, *T_beg_*. Growth is restricted when the temperature falls below a minimum threshold (*T_min_*). The growth rate increases linearly up to the first optimum temperature (*T_opt_*_1_), remains constant between *T_opt_*_1_ and the second optimum temperature (*T_opt_*_2_), and then decreases linearly until the maximum limiting temperature (*T_max_*), where growth ceases.

Soil Moisture: Similarly to temperature, the impact of soil moisture on tree growth is quantified using a piecewise function with parameters *W_min_*, *W_opt_*_1_, *W_opt_*_2_, *and W_max_*. The daily soil water content (*dW*) is determined by a water balance equation accounting for precipitation, *f* (*P*), transpiration (*Er*), and runoff (*Q*). The actual precipitation (*P*) is multiplied by a coefficient (*k*_1_) to determine soil infiltration:

*dW* = *f* (*P*) − *Er* − *Q* (2)

*f* (*P*) = min [*k*_1_ × *P*, *P*_*max*_] (3)

The VS model is a multi-parametric model that presents significant challenges in determining the appropriate parameter values to create a simulated chronology that closely matches the observed tree-ring chronology [34]. In this study, certain parameters were established based on observed data, including the coefficient for water infiltration from soil (*k_r_*), the depth of the root system (*D_root_*), the maximum daily precipitation entering the soil (*P_max_*), and the cumulative temperature for growth initiation (*T_beg_*) [29]. Other critical parameters, including soil moisture (*W_min_*, *W_opt_*_1_, *W_opt_*_2_, *W*_max_), temperature (*T_min_*, *T_opt_*_1_, *T_opt_*_2_, *T_max_*), and coefficients (*K*_1_, *K*_2_, and *K*_3_), were calibrated through an iterative process of refinement and comparison with the observed chronology. Initial values for these parameters were informed by previous studies conducted in similar regions [16]. The chronology was divided into two periods: calibration (1958–1984) and verification (1985–2011). The Pearson correlation coefficient between simulated and observed chronologies served as the metric for evaluating the accuracy of the VS model simulations.

### 2.5. Data Analysis and Statistics

To examine the relationship between tree growth and climate, correlation analysis was conducted by using STD chronology (observed tree-ring index). The correlation coefficients were calculated between the STD chronology and monthly regional climate data from 1958 to 2011. To consider the impact of climatic conditions on tree growth for both the current and previous years [35], the correlation analysis spanned a 14-month period from the previous August to the current October.

To assess the growth rate during different periods of the growing season, we computed the daily mean growth rates (*Gr*, *GrE*, *GrT*, and *GrW*) between years. Furthermore, to understand the impact of climatic variability on ring width formation, we compared intra-annual growth variations between years with wide and narrow rings, defined as one standard deviation above or below the mean ring width, respectively.

Additionally, both simulated and observed tree-ring width chronologies were correlated with regional gridded annual mean precipitation data from CRU TS 4.05 (http://climexp.knmi.nl, accessed on 18 February 2024; range of 34°–38° N, 101°–106° E) for the period from the previous August to the current July. This analysis aimed to validate the accuracy of the simulation and to explore the influence of climate on tree growth within the study region.

## 3. Results

### 3.1. Tree-Ring and Climate Responses

**Figure 4:**
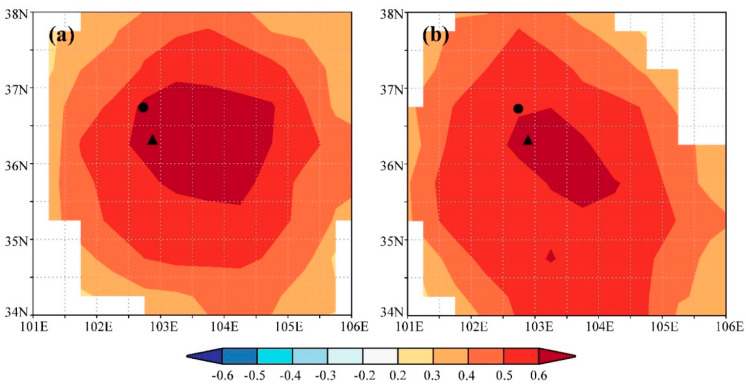
Spatial correlations between gridded precipitation from the previous August to the current July of CRU TS 4.05 and the (**a**) observed and (**b**) simulated tree-ring width chronology. Insignificant correlations (*p* > 0.05) are masked. The triangle and circle indicate the sampling site and Minhe meteorological station, respectively.

We found significant positive correlations between the ring-width index and precipitation in the previous August (*p* < 0.05) and the current July (*p* < 0.01). In contrast, there were significant negative correlations between the ring-width index and temperature in the previous September and the current May, June, and August (*p* < 0.05). The strength of these correlations was more significant when considering the annual mean precipitation, with the correlation coefficient between tree growth and precipitation from the previous August to the current July exceeding 0.6 (*p* < 0.01). Similarly, tree growth exhibited a significant negative correlation with the annual mean temperature for the same period. These results suggest that water availability, as indicated by precipitation, likely plays a crucial role in tree growth of the study region. Spatial correlation analysis further confirmed these findings, showing strong positive correlations between the tree-ring width index and precipitation from the previous August to the current July across extensive areas of the study region (Figure 4a), highlighting the significance of water availability in influencing tree growth across a wide geographic extent.

### 3.2. Process Model Analysis

**Figure 5:**
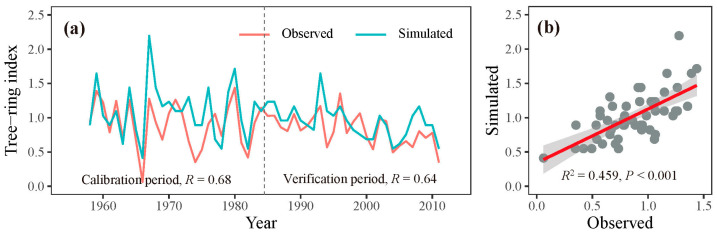
(**a**) Simulated (blue lines) and observed (red lines) chronologies for the calibration (1958–1984) and verification (1985–2011) periods. (**b**) The relationship between the observed and simulated chronologies during 1958–2011.

Through iterative refinement and correlation comparison between simulated and observed chronologies, we identified the most appropriate physiological parameters for this study (Table 2). Using these optimized parameters, we observed a significant correlation (r = 0.68, *p* < 0.001, n = 27) between the observed and simulated chronologies during the calibration period 1958–1984. A similar level of agreement (r = 0.64, *p* < 0.001, n = 27) was found during the verification period 1985–2011 (Figure 5a). When combining these two periods, the simulated chronology explained 45.9% of the variance in the observed chronology (Figure 5b). Additionally, both the observed and simulated chronologies exhibited strong positive correlations with precipitation from the previous August to the current July across extensive regions of the study region (Figure 4). These results underscore the effectiveness of the process-based VS model in simulating tree-ring formation and in capturing large-scale patterns of tree growth responses to climate change in semi-arid conditions, such as those in the eastern Qilian Mountains.

### 3.3. Model Outputs of Phenology and Growth Rate of Trees

**Figure 6:**
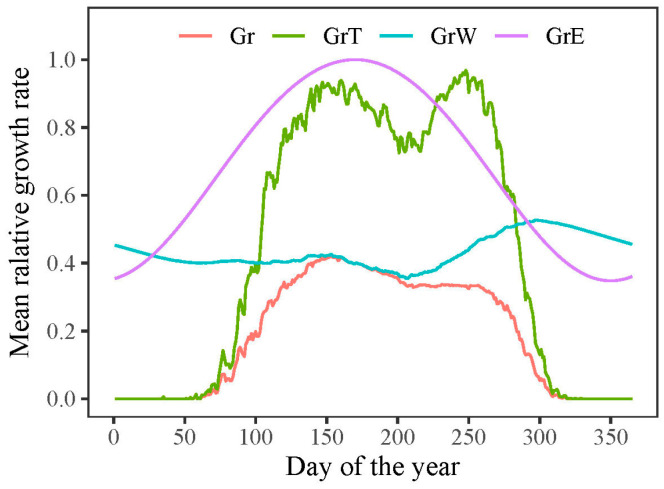
Simulated radial growth rates dependent on solar radiation (*GrE*), temperature (*GrT*), soil moisture (*GrW*), and the combined effect of all factors (*Gr*) during 1958–2011.

According to the VS model, the overall growth rate (*Gr*) of *P. tabuliformis* was primarily impacted by the lower growth rates associated with temperature (*GrT*) or soil moisture (*GrW*). The model outputs indicated that both the onset and end of radial growth in *P. tabuliformis* were determined by temperature-dependent growth rates (Figure 6). The mean onset and end of growth in *P. tabuliformis* occurred on 13 April (DOY 103) and 11 October (DOY 284), respectively, resulting in an average growing season of 182 days during the study period (1958–2011).

**Figure 7:**
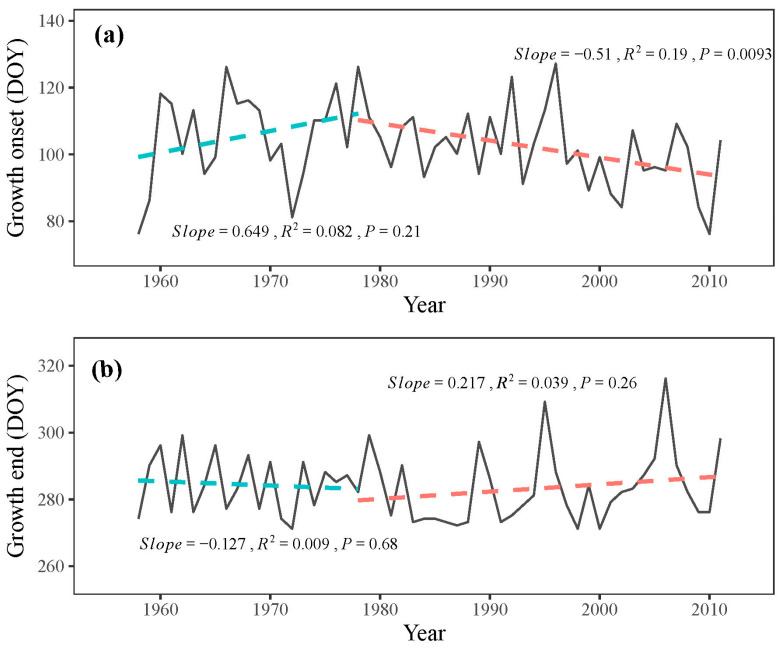
Simulated (**a**) onset and (**b**) end of growth (solid line) with their respective trends (dashed line) during 1958–2011.

Although not significant (*p* = 0.21), the onset of the growing season exhibited a delayed trend from 1958 to 1978, with a delay rate of 6.5 days per decade during these two decades (Figure 7a). However, after 1978, the onset of the growing season occurred significantly earlier (*p* < 0.05), with an advancement rate of 5.1 days per decade (Figure 7a). No distinct trend was observed at the end of the growing season throughout the study period, although a non-significant advancement trend (*p* = 0.68) from 1958 to 1978 and a delayed trend (*p* = 0.26) from 1978 to 2011 was found, respectively (Figure 7b). These simulated results suggest an extension of the growing season in *P. tabuliformis* after 1978.

**Figure 8:**
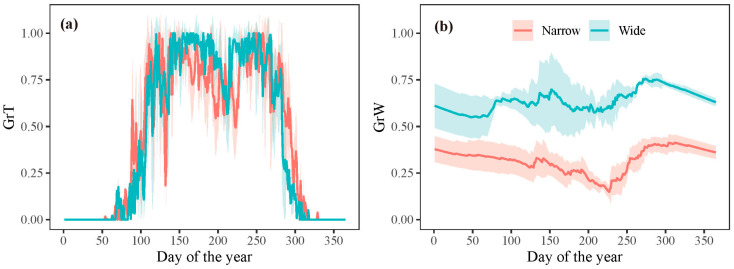
Simulated mean growth rate dependent on (**a**) temperature and (**b**) soil moisture for years with wide and narrow rings, respectively. The shaded areas represent plus/minus one standard deviation.

However, the extension of the growing season did not translate into an increased growth rate. The model outputs revealed that once the growth commenced, the growth rate was predominantly determined by water conditions (*GrW*) and remained consistent throughout the growing season (Figure 6). When analyzing intra-annual tree growth differences between years with wide and narrow rings using the model, no significant difference was found in the average temperature-dependent growth rate between wide and narrow rings (Figure 8a). This result indicates that temperature was not the primary limiting factor for the formation of wide or narrow rings. However, the mean relative growth rate due to soil moisture was significantly lower in narrow rings than in wide rings during the growing season, highlighting the crucial role of water availability in tree growth during this period (Figure 8b).

## 4. Discussion

### 4.1. Impacts of Precipitation on Tree Growth

The tree-ring index exhibited significant positive correlations with monthly precipitation, indicating that water availability is a key determinant of *P. tabuliformis* growth in the eastern Qilian Mountains. This semi-arid region, characterized by steep slopes, receives low annual precipitation (averaging 350 mm over the past six decades), which is insufficient to meet the trees’ water demands [36]. The higher temperatures in the region likely contributed to increased potential evapotranspiration, further decreasing soil moisture. This is supported by the significant negative correlations between the tree-ring index and summer temperatures during the current growing season, which underscore the importance of water availability for tree growth in this region. Therefore, moisture availability is identified as the primary growth-limiting factor in this area.

The significant positive correlation with precipitation and negative correlation with temperature in the previous August suggests that carbohydrates produced at the end of the previous growing season may contribute to the current year’s growth. This is supported by the monitoring study of photosynthates in the same species within our study region, which reported high photosynthetic rates in August and September at the end of the growing season [37]. Dendroclimatic studies in the eastern Qilian Mountains have also indicated the sensitivity of ring widths to moisture availability in various tree species, including *Picea crassifolia* [38,39], *Picea wilsonii* [40], and *Juniperus przewalskii* [41]. These findings are further supported by intra-annual monitoring studies in the region, which found that the intra-annual radial growth of trees is mainly correlated with the growing season precipitation [29,30,42].

### 4.2. Effects of Temperature on Phenology of Tree Growth

The VS model outputs provide valuable insights into the growth phenology over the study period. The simulated mean growth onset (13 April, DOY 103) during 1958–2011 aligns with the same period (mid-April) as we monitored in the same region in *P. tabuliformis* during the 2012–2021 growing seasons [29]. Thus, the VS model provides significant insights into the onset of tree growth during the past decades. However, the simulated mean growth end (11 October, DOY 284) is half a month later than the observed data (late September) [29]. This suggests that the VS model more accurately simulates growth onset than cessation. Previous studies have also highlighted better performance of the VS model in predicting growth onset than growth end [17,18,20], possibly due to a lack of modules accounting for carbon storage processes at the end of the growing season [18]. Additionally, the model may not fully capture the impact of water availability on the end of the growing season in arid or semi-arid regions, resulting in an overestimation of the timing of growth end [29]. Despite these limitations, the VS model demonstrates trends similar to those observed in phenology and is widely used for assessing long-term growth phenologies [14].

The model indicates that temperature is the primary factor determining the growth onset in *P. tabuliformis* over the eastern Qilian Mountains, with a minimum threshold temperature of 6 °C. This is consistent with our monitoring results that identify temperature as the main factor determining the onset of cell production in coniferous species over the eastern Qilian Mountains, including *P. tabuliformis* [29,30]. This finding is also in line with studies in temperate and boreal forests, where temperature significantly impacts the onset of tree growth [8,30,43]. For example, the mean threshold temperature for the onset of radial growth in *J. przewalskii* in the eastern Qilian Mountains is 6.1 °C, similar to findings at treeline in the eastern Italian Alps [44,45].

The model also suggests that temperature controls the end of radial tree growth, with the growth terminating as temperatures decrease to the threshold. This aligns with the monitoring studies in cold regions where temperature plays an important role in the end tree growth [8,46]. However, monitoring studies in the eastern Qilian Mountains indicate that summer drought conditions, driven by high temperature and soil moisture deficits, may determine the end of wood formation before the temperature decreases to the threshold [29,30]. Cambial cell division in a dry environment is closely related to water potential [47,48,49], and water deficit may lead to a decrease in cell turgor, resulting in a termination in cell division and enlargement toward the end of the growing season [50]. Therefore, further research is necessary to better understand the limitations of the VS model and to develop the model to account for the complex interactions between water availability and tree growth in semi-arid regions.

The VS model provides comprehensive growth phenologies for the entire simulated period, surpassing the limitations of radial growth monitoring studies that are typically based on one or a few years [14]. This enables us to analyze the changing trends in phenologies over recent decades and estimate the potential advancement of tree growth under future climate change. Our study indicates a delayed growth onset in the initial decades (1958–1978), followed by advancement in later years (1978–2011), corresponding with observed temperature trends. These findings reinforce the critical role of temperature in influencing the onset of radial growth in the eastern Qilian Mountains.

Predictions based on this study suggest that if temperatures continue to rise at the current rate in the study region (0.45 °C decade^−1^), the onset of radial tree growth in *P. tabuliformis* will occur approximately 5.1 days earlier per decade, while the end of tree growth will be delayed by approximately 2.2 days per decade. These projections are similar to those for *J. przewalskii* in the same region, where the onset advanced by 3.1 days and the end by 1.8 days per decade [30]. However, the uncertainty of the simulation regarding the growth end must be acknowledged, as the model’s consideration of the influence of water on the end of tree growth requires further refinement.

### 4.3. Influence of Water Availability on Intra-Annual Tree Growth

Our study revealed that an extended growing season, driven by rising temperatures, did not necessarily enhance radial growth. Instead, the growth rate of *P. tabuliformis* remained dependent on water conditions in the eastern Qilian Mountains. The significant difference in model outputs between years with wide and narrow rings further emphasizes the importance of water availability over temperature in determining tree growth rates in our study region. This is supported by the correlations between the observed STD chronology and climatic responses, indicating that tree growth is influenced by precipitation in July and the previous August. Similar findings have been reported in the region for different species based on microcoring studies, which emphasize the critical role of precipitation during the growing season in influencing growth rate [30,42]. Consistently, both intra-annual [51,52] and inter-annual [41,53,54] tree-ring growth studies have demonstrated a positive correlation between early summer precipitation and drought conditions on the northeastern Tibetan Plateau, suggesting that water availability is the primary factor influencing growth rate and wood production in alpine semi-arid environments.

Spatial correlation analysis revealed strong positive correlations between both the observed and simulated tree-ring width chronologies and precipitation from the previous August to the current July across the study region, highlighting the significance of water availability during this period for regional tree growth. However, when comparing the two correlation fields, the observed tree-ring index exhibited a higher correlation coefficient and a broader correlation field than the simulated index, suggesting that the observed index contains more climate information than the simulated index. This result was not found in previous simulations in *J. przewalskii* near the study region [16,55]. This discrepancy may arise because the VS model only considers climate information for the current year, omitting previous year influences, which could explain why the simulated index does not fully reflect climate information present in the observed index.

## 5. Conclusions

The process-based VS model was employed to simulate the phenology and growth dynamics of *P. tabuliformis* in the eastern Qilian Mountains using temperature and precipitation data from meteorological stations. Our study demonstrates that the simulated tree-ring index closely aligns with the observed tree-ring chronology, indicating the accuracy of the simulations in reflecting the climatic influences on tree growth in *P. tabuliformis* in this semi-arid region. The outputs from this model agree with the statistical relationships between tree-ring growth and climate factors. The findings suggest that growth phenologies are predominantly determined by threshold temperatures, while the growth rate is primarily influenced by soil moisture availability during the growing season. Notably, temperature was not found to have a significant impact on the rate of radial growth. These insights are in line with previously observed growth patterns for *P. tabuliformis* and other species within the same region. However, the VS model does not account for the effects of climate conditions from the previous growing season on the subsequent year’s tree growth, which could lead to discrepancies in the simulation results. Future enhancements to the model should incorporate this factor to improve the precision and reliability of growth simulations.

## Figures and Tables

**Table 1:** Statistical characteristics and common interval analyses results of the STD chronology.

Statistical Parameters	Value
Mean sensitivity (MS)	0.507
Inter-series correlation	0.827
Mean segment length (yr)	84.3
Absent rings (%)	0.742
All series Rbar	0.613
Standard deviation	0.160
Expressed population signal (EPS)	0.978
Signal-to-noise ratio (SNR)	44.441
Year of SSS > 0.85/yr (cores)	1879 (4)

**Table 2:** Model parameters used in this study.

Parameter	Description (Units)	Value
*T_min_*	Minimum temperature for tree growth (°C)	6
*T_opt_*_1_	Lower limit of optimal temperatures (°C)	14
*T_opt_*_2_	Upper limit of optimal temperatures (°C)	18
*T_max_*	Maximum temperature for tree growth (°C)	30
*W_min_*	Minimum soil moisture for tree growth (*v*/*v*)	0.04
*W_opt_*_1_	Lower limit of optimal soil moisture (*v*/*v*)	0.29
*W_opt_*_2_	Upper limit of optimal soil moisture (*v*/*v*)	0.8
*W_max_*	Maximum soil moisture for tree growth (*v*/*v*)	0.9
*T_beg_*	Temperature sum for initiation of growth (°C), period = 10 days	55
*D_root_*	Depth of root system (mm)	1000
*P_max_*	Maximum daily precipitation for saturated soil (mm)	20
*k*_1_	Fraction of precipitation penetrating soil	0.86
*k*_2_	First coefficient for calculation of transpiration (mm/day)	0.12
*k*_3_	Second coefficient for calculation of transpiration (1/degree)	0.176
*k_r_*	Coefficient for water infiltration from soil	0.002

## Data Availability

Data are available from the corresponding author on reasonable request.
